# Screening for Older Emergency Department Inpatients at Risk of Prolonged Hospital Stay: The Brief Geriatric Assessment Tool

**DOI:** 10.1371/journal.pone.0110135

**Published:** 2014-10-15

**Authors:** Cyrille P. Launay, Laure de Decker, Anastasiia Kabeshova, Cédric Annweiler, Olivier Beauchet

**Affiliations:** 1 Department of Neuroscience, Division of Geriatric Medicine, UPRES EA 4638, UNAM, Angers University Hospital, Angers, France; 2 Department of Geriatrics, EA 1156–12, Nantes University Hospital, Nantes, France; 3 Robarts Research Institute, Schulich School of Medicine and Dentistry, the University of Western Ontario, London, Ontario, Canada; 4 Biomathics, Paris, France; Hospital Universitario de Getafe, Spain

## Abstract

**Background:**

The aims of this study were 1) to confirm that combinations of brief geriatric assessment (BGA) items were significant risk factors for prolonged LHS among geriatric patients hospitalized in acute care medical units after their admission to the emergency department (ED); and 2) to determine whether these combinations of BGA items could be used as a prognostic tool of prolonged LHS.

**Methods:**

Based on a prospective observational cohort design, 1254 inpatients (mean age ± standard deviation, 84.9±5.9 years; 59.3% female) recruited upon their admission to ED and discharged in acute care medical units of Angers University Hospital, France, were selected in this study. At baseline assessment, a BGA was performed and included the following 6 items: age ≥85years, male gender, polypharmacy (i.e., ≥5 drugs per day), use of home-help services, history of falls in previous 6 months and temporal disorientation (i.e., inability to give the month and/or year). The LHS in acute care medical units was prospectively calculated in number of days using the hospital registry.

**Results:**

Area under receiver operating characteristic (ROC) curves of prolonged LHS of different combinations of BGA items ranged from 0.50 to 0.57. Cox regression models revealed that combinations defining a high risk of prolonged LHS, identified from ROC curves, were significant risk factors for prolonged LHS (hazard ratio >1.16 with P>0.010). Kaplan-Meier distributions of discharge showed that inpatients classified in high-risk group of prolonged LHS were discharged later than those in low-risk group (P<0.003). Prognostic value for prolonged LHS of all combinations was poor with sensitivity under 77%, a high variation of specificity (from 26.6 to 97.4) and a low likelihood ratio of positive test under 5.6.

**Conclusion:**

Combinations of 6-item BGA tool were significant risk factors for prolonged LHS but their prognostic value was poor in the studied sample of older inpatients.

## Introduction

Older adults are the fastest increasing group of patients admitted to hospital, often through emergency departments (ED) [1], [2]. Compared to younger inpatients, multimorbidity and related-disabilities leading to a high disease burden characterize the older inpatients group [3]–[5]. Because hospital is largely configured for single acute disease rather than multimorbidity and related disability, age-related high burden disease is one of the main challenges faced by hospitals [6]. Thus, assessing and addressing the needs of the growing number of older inpatients with a greater range of acute and chronic diseases causing disability but not mortality is mandatory [6]–[8].

Adapted care plan of older inpatients are based on multidimensional, interdisciplinary diagnostic process to determine the medical, psychological, and functional capabilities called comprehensive geriatric assessment (CGA) [3]. CGA allows a coordinated and integrated plan for treatment, and thus may prevent complicated medical pathway characterized, for instance, by prolonged length of hospital stay (LHS) [4], [3], [9]. Early screening of older inpatients at risk of prolonged LHS based on CGA approach is the first step of an adapted care plan of older inpatients because it improves the medical decision-making process, and thus the care pathway [4], [10].

Benefits of CGA have been confirmed through a meta-analysis reporting a reduction of admissions and readmissions to hospital [11]. Recently, it has been reported that an early intervention made by geriatricians of a mobile geriatric team (MGT) using a 6-item brief CGA (BGA) reduced LHS in older inpatients [12]. The 6-item BGA has been developed in response to the difficulty of systematic implementation of CGA in daily practice for every older inpatient admitted to ED [4], [13], [14]. It has been shown that CGA cannot be applied to every older inpatient, and that the best compromise could be the use of two-step approach [15]. The first step is the screening of older inpatients at high risk of adverse outcomes such as prolonged LHS by non-geriatricians using a brief and accurate screening tool applicable during clinical practice, and the second step is a CGA by geriatricians with a diagnostic and therapeutic purpose [15], [16].

We recently showed that different combinations of the 6-item BGA tool (i.e., age ≥85 years, gender male, polypharmacy, non-use of home services, history of falls and cognitive decline) were risk factors for prolonged LHS in a cohort of 424 older patients admitted to acute care medical units following ED discharge [16], [17]. Among these different combinations, history of falls and cognitive decline were major predictors for prolonged LHS. We also reported that the maximum number of items to predict prolonged LHS was 4. Indeed, the combination of a history of falls, male gender, cognitive impairment and age under 85 years, identified older patients at highest risk of prolonged LHS. These results allowed the identification of 3 levels of risk of prolonged LHS (i.e., high, intermediate and low) according to different combinations of the BGA 6 items. The aims of this study were 1) to confirm that combinations of BGA items were significant risk factors for prolonged LHS among geriatric patients hospitalized in acute care medical units after their admission to the ED; and 2) to determine whether these combinations of BGA items could be used as a prognostic tool of prolonged LHS.

## Methods

### Recruitment of participants

**Figure 1:**
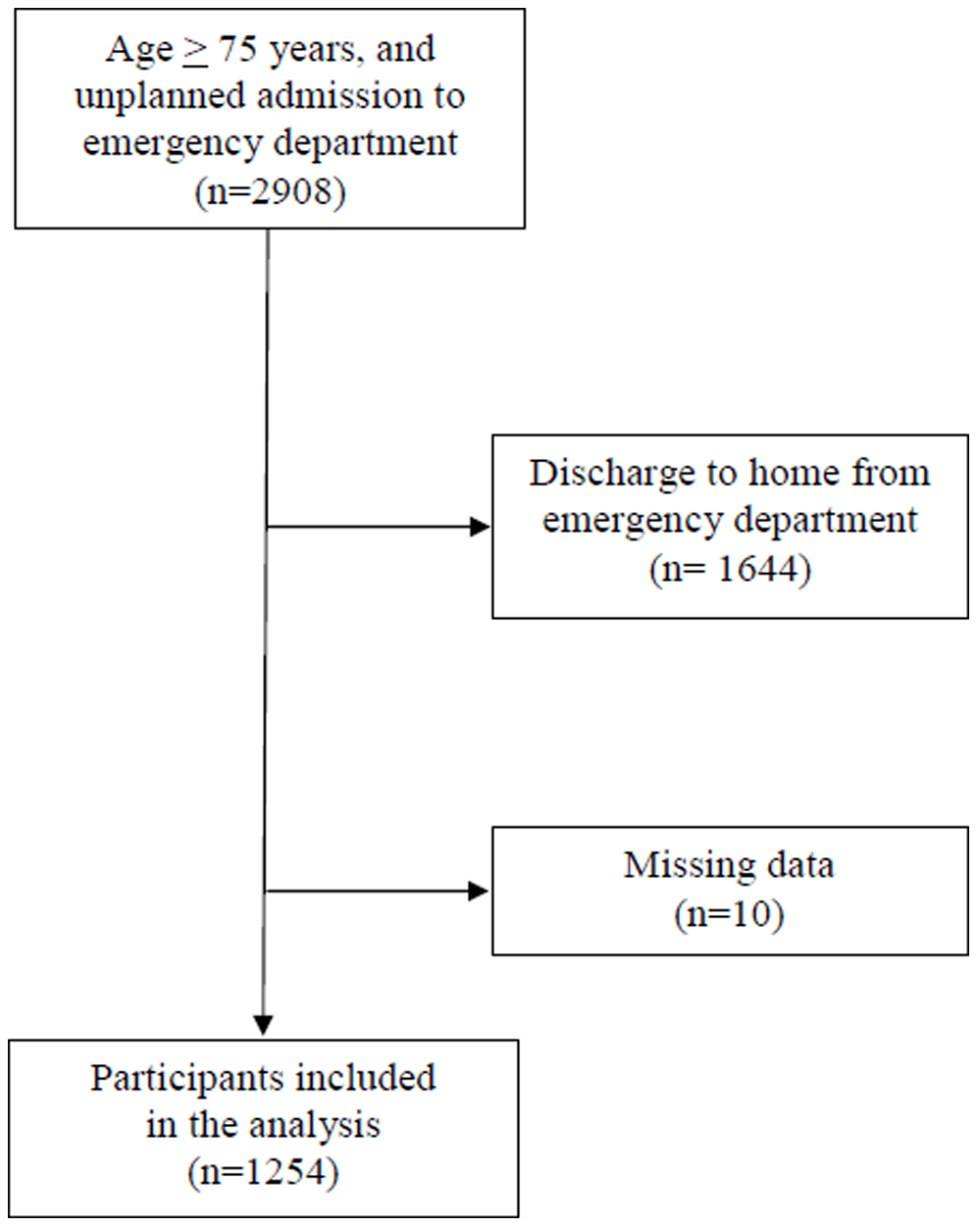
Flow chart showing the selection of participants included in the analysis.

Between January and December 2012, 2908 older adults aged 75 years and over were admitted to the ED of Angers University Hospital, France. Among them, 1264 (43.5%) were discharged in acute care medical units. None of them declined to be part of the study. Inclusion criteria were: unplanned admissions in ED followed by discharge in acute care medical units, age of 75 years and over, not identified as treatment-limiting decision and willingness to participate. A total of 10 participants have missing data and were excluded form the analysis. In final, 1254 participants (mean age ± standard deviation, 84.9±5.9 years; 59.3% female) were included in the study (Figure 1).

### Assessment of participants

The 6-item BGA was performed upon the admission to ED. It was composed of the following items: age coded as a binary variable (i.e.,≥ or <85 years), gender, polypharmacy defined as ≥5 drugs per day, use of formal and/or informal home-help services coded as a binary variable (i.e., yes or no), history of falls in previous 6 months coded as a binary variable (i.e., yes or no), and temporal disorientation defined as the inability to give the month and/or year coded as a binary variable (i.e., yes or no). We chose these items because each of them has been separately associated with a prolonged LHS and because we previously reported that their combinations predicted prolonged LHS [16]–[20]. The place of life (i.e., home versus institution) and the reasons for admission to ED were also recorded because these parameters have both been associated with prolonged LHS [18]–[20]. Reasons for admission were categorized using two complementary approaches. First, we distinguished acute organ failure versus non-acute organ failure. Second, we specified the nature of the acute organ failure in four subgroups based on the prevalence of related diseases in our sample of participants: 19.5% (n = 156) cardio-vascular diseases, 18.4% (n = 147) respiratory diseases, 16.2% (n = 129) digestive diseases, 12.0% (n = 96) neuropsychiatric diseases and 33.9% (n = 271) other acute diseases. In addition, the use of psychoactive drugs including benzodiazepines, antidepressants or neuroleptics was noted.

### Definition of endpoints

The risk of prolonged LHS was estimated using two consecutive strategies. First, *a priori* combinations of BGA items as previously reported to determine 3 levels of risk of prolonged LHS [16], [17]. High risk of prolonged LHS was defined by the combination of cognitive decline + history of falls. Intermediate risk was defined by the finding of cognitive decline, or history of falls, or the combination of age ≥85years + male gender + polypharmacy + no use of home services. Low risk of prolonged LHS was defined by the combination of 3 items or less among age ≥85years, male gender male, polypharmacy, and no use of home services. No information was provided to the physicians in charge of the patients. Second, we explored all other possible combinations of BGA items using the receiver operating characteristic (ROC) curve. A new classification into three levels of risk of prolonged LHS was therefore built using areas under ROC separated into three parts (i.e., highest, intermediate and lowest values). The three best combinations for each part were used to define the risk of prolonged LHS. The LHS was prospectively calculated using the administrative registry of the University Hospital and corresponded to the delay in days between the first day of admission to ED and the last day of hospitalization in the acute care medical units. Prolonged LHS was defined as being in the highest tertile of LHS (i.e.,>13 days).

### Statistical analysis

First, the participants' baseline characteristics were summarized using means and standard deviations or frequencies and percentages, as appropriate. Second, areas under ROC curve of prolonged LHS was calculated for each item and all possible combinations. Third, univariate and multiple Cox regression models were performed to examine the association between prolonged LHS (dependent variable) and the different combinations of BGA items (independent variables) stratified in three risk-levels (low, intermediate, high). For multiple Cox regression models, two types of models were distinguished: those using low-risk level of prolonged LHS as reference and the others. In both cases, there was an adjustment on the place of life and the reasons for admission to ED. Two kind of adjustment were done for reasons of admission using either acute organ failure as independent variable or the nature of acute organ failures (i.e., cardio-vascular diseases, respiratory diseases, digestive diseases, neuropsychiatric diseases and other diseases). Exponential of negative coefficients were calculated for each model. The model produced a survival function that provided the probability of discharge of hospital at a given time for the features supplied for independent variables. Fourth, we studied the elapsed time to discharge from acute care medical units by survival Kaplan-Meier curves and log-rank test. Because only discharge to acute care medical unit was examined, the analysis was censored to 21 days. Fifth, sensitivity, specificity, positive (PPV) and negative (NPV) predictive values, likelihood ratios of positive (LR+) and negative (LR-) tests of each BGA item and all possible combinations of prolonged LHS were calculated. P-values <0.05 were considered statistically significant. All statistics were performed using SPSS (version 19.0; SPSS, Inc., Chicago, IL) and RStudio.

### Standard Protocol Approvals, Registrations, and Participant Consents

The study was conducted in accordance with the ethical standards set forth in the Helsinki Declaration (1983). All participants recruited in this study provided a verbal informed consent because the study did not change the usual clinical practice. The verbal informed consent was obtained from the patients themselves in the presence of their trusted person, usually a family member, who helped them to make decision. The participant consent was recorded in the digital file of patients. The entire study protocol and the consent procedure were approved by the Ethical Committee of Angers, France.

## Results

The baseline characteristics of participants are presented in Table 1. More than half of patients were aged 85 years and over. A total of 74.1% took 5 and over different drugs per day. Nearly one third had a history of falls in the past 6 months or had temporal disorientation. One quarter of participants were isolated, and more than two thirds lived at home. A total of 63.3% patients were admitted to ED for an acute organ failure. The main causes of acute organ failure were cardiovascular diseases (19.5%), respiratory diseases (18.4%), digestive diseases (16.2%) and neuropsychiatric diseases (12.0%). The mean LHS was around 9 days. A total of 157 patients (12.5%) died during hospitalization. They were older than those who did not die (mean age ± standard deviation, 86.2±5.4 years versus 84.7±5.9 with P = 0.004) and there were fewer women in this group (49.0% versus 60.4%, P = 0.007). In addition, they had a longer LHS compared to those who did not die (11.3±10.5 days versus 8.4±8.1 days with P<0.001).

**Table 1:** Baseline characteristics of participants (n = 1254).

Characteristics	Value	[95% confidence interval]
Age (years)		
Mean ±SD	84.9±5.9	[84.6–85.3]
≥85 years	671 (53.5)	[50.7–56.3]
Male gender, n (%)	511 (40.7)	[38.0–43.5]
Number of drugs daily taken		
Mean ±SD	6.7±3.2	[6.6–6.9]
≥5, n (%)	929 (74.1)	[71.7–76.5]
Use of psychoactive drugs*, n (%)	646 (51.1)	[48.8–54.3]
History of falls during the past 6 months, n (%)	468 (37.3)	[34.6–40.0]
Temporal disorientation†, n (%)	415 (33.1)	[30.5–35.7]
Non-use of formal and/or informal home services‡	310 (24.7)	[22.3–27.1]
Living at home	875 (69.7)	[67.2–72.3]
Acute organ failure as reason for admission to Emergency Department, n (%)	799 (63.3)	[61.0–66.4]
Cardio-vascular diseases, n (%)	156 (19.5)	[16.8–22.3]
Respiratory diseases, n (%)	147 (18.4)	[15.7–21.1]
Digestive diseases, n (%)	129 (16.2)	[13.6–18.7]
Neuropsychiatric diseases, n (%)	96 (12.0)	[9.8–14.3]
Other diseases, n (%)	271 (33.9)	[30.6–37.2]
Length of hospital stay (days), mean ±SD	8.8±8.4	[8.3–9.2]

SD: standard deviation.

*: Use of benzodiazepines or antidepressants or neuroleptics.

† : Inability to give the month and/or year.

‡ : Formal (i.e., health and/or social professional) or informal (i.e., family and/or friends).

Among BGA items, the best predictor of prolonged LHS was temporal disorientation, this item having the highest value of ROC curve (0.55) (Table 2). The combination ‘history of falls’ plus ‘temporal disorientation’, defining the *a priori* high risk of prolonged LHS identified in our previous publication [16], had the highest value of ROC curve (0.56). Its sensitivity and PPV were low (18.46% and 29.57) but the specificity and NPV were high (86.30% and 77.25). The LR+ and LR- were calculated at 1.35 and 0.95. Compared to this *a priori* classification, new combinations defining high risk of prolonged LHS had highest values of ROC curve. The sensitivity of all combinations of this level of risk was low under 13.0% but the specificity was high above 90.0%. In addition, the highest LR+ of 5.50 was shown for a combination involving ‘age ≥85 years’ plus ‘polypharmacy’ plus ‘non-use of home-help services’.

**Table 2:** Area under Receiver operating characteristic curve and prognostic values of brief geriatric assessment items and their combinations* for prolonged length of hospital stay† (n = 1254).

	Area under ROC	Sensitivity(%)	Specify(%)	PPV (%)	NPV (%)	LR+	LR-	Number of individuals classified			
								TP	FP	FN	TN
6 items of the brief geriatric assessment, considered separately											
Age≥85 years	0.50	53.02	46.34	23.55	75.99	0.99	1.01	158	513	140	443
Male gender	0.50	40.94	59.31	23.87	76.31	1.00	1.00	122	389	176	576
Polypharmacy‡	0.51	76.17	26.57	24.43	78.15	1.04	0.90	227	702	71	254
Non-use of home help services§	0.52	27.85	76.26	26.77	77.22	1.17	0.95	83	227	215	729
History of falls¶	0.52	39.93	63.49	25.43	77.23	1.09	0.95	119	349	179	607
Temporal disorientation#	0.55	40.94	69.35	29.40	79.02	1.34	0.58	122	293	176	663
Combinations of the 6 items of the brief geriatric assessment **											
*A priori* combinations††											
Low risk											
Male + polypharmacy‡ + non-use of home services§	0.54	9.40	91.95	26.67	76.50	1.17	0.99	28	77	270	879
Age≥85 years + polypharmacy‡ + non-use of home services§	0.53	9.06	94.46	33.75	76.92	1.63	0.96	27	53	271	903
Age≥85 years + male + non-use of home services§	0.52	3.69	96.13	22.92	76.20	0.95	1.00	11	37	287	919
Intermediate risk											
History of falls	0.52	39.93	63.49	25.43	77.23	1.09	0.95	119	349	179	607
Temporal disorientation#	0.55	40.94	69.35	29.40	79.02	1.34	0.58	122	293	176	663
Age ≥85years + male + polypharmacy‡ + non-use of home services§	0.54	2.35	97.38	21.88	76.19	0.90	1.00	7	25	291	931
High risk											
History of falls + temporal disorientation#	0.56	18.46	86.30	29.57	77.25	1.35	0.95	55	131	243	825
Newly identified combinations											
Low risk											
Polypharmacy‡ + non-use of home help services§	0.53	17.79	84.21	25.98	76.67	1.13	0.98	53	151	245	805
Polypharmacy‡ + history of falls	0.53	31.88	75.00	28.44	77.93	1.28	0.91	95	239	203	717
Age≥85 years + polypharmacy‡ + history of falls	0.53	17.79	83.89	25.60	76.60	1.10	0.98	53	154	245	802
Intermediate risk											
Age≥85 years + polypharmacy‡ + temporal disorientation#	0.56	15.44	86.72	26.59	76.69	1.16	0.98	46	127	252	829
Male + polypharmacy‡ + temporal disorientation#	0.56	12.08	92.26	32.73	77.10	1.56	0.95	36	74	262	882
Age≥85 years + male + polypharmacy‡ + temporal disorientation#	0.56	5.37	95.26	28.57	76.46	1.28	0.99	16	40	232	915
High risk											
History of falls + temporal disorientation# + Polypharmacy‡	0.57	12.75	91.32	31.40	77.05	1.47	0.96	38	83	260	873
History of falls + temporal disorientation# + Polypharmacy‡ + male	0.57	5.03	96.55	31.25	76.53	1.46	0.98	15	33	283	923
Age≥85 years + polypharmacy‡ + non-use of home help services§	0.57	4.03	99.27	63.16	76.84	5.50	0.97	12	7	286	949

ROC: Receiver operating characteristic curve; PPV: Positive predictive value; NPV: Negative predictive value; LR+: Likelihood ration of positive test; LR-: Likelihood ration of negative test; TP: True positive; FP: False positive; TN: True negative; FN: False negative;

*: Only three best models (i.e., highest value of ROC) by level (i.e., Low, intermediate and high risk) are shown;

† : Defined being in the highest tertile of length of hospital saty (i.e.,>13 days);

‡ : Defined by a number of drugs taken per day above 4;

§ : Living alone without using any formal or informal home services and social help;

¶ : during the past 6 months;

# : Inability to give the month and/or year;

**: Only combination involving at least 10 participants were considered;

†† : Combinations described in previous published study (Beauchet et al. J Emerg Med. 2013; 45: 739–45).

Univariate and Cox regression models adjusted on acute organ failure and place of life showed that patients classified *a priori* with low risk of prolonged LHS had indeed a significantly shorter LHS (unadjusted Hazard Ratio [HR]  = 0.82 with P = 0.001, and adjusted HR = 0.80 with P<0.001), whereas those with high risk of prolonged LHS had a longer LHS (unadjusted HR = 1.27 with P = 0.003, adjusted HR = 1.29 with P = 0.002 and fully adjusted HR = 1.44 with P<0.001) (Table 3). In addition, fully adjusted Cox model (i.e., using the group at low risk of prolonged LHS as reference) shown that patients in intermediate risk group (HR = 1.20 with P = 0.006) had also prolonged LHS. Classification of risk of prolonged LHS based on newly identified combinations underscored that only high risk level predicted a prolonged LHS (unadjusted HR = 1.40 with P<0.001, adjusted HR = 1.43 with P<0.001 and fully adjusted HR = 1.47 with P<0.001). As shown in Table 4, adjustment for the nature of acute organ failures did not alter our results. Indeed, intermediate risk and high risk of prolonged LSH predicted effectively a prolonged LSH with *a priori* combinations (HR>1.15 and P<0.015), whereas only high risk level predicted prolonged LSH with new combinations (HR>1.46 and P<0.001).

**Table 3:** Cox regression models showing the association between the length of hospital stay (dependent variable) and combinations of brief geriatric assessment items (independent variables) separated into three risk-levels (i.e., low risk, intermediate risk, and high risk of prolonged length of hospital stay) (n = 1254).

Combinations of the 6-item brief geriatric assessment, stratified in three levels of risk (low, intermediate, high)	HR [95% CI] (P-Value)		
	Model 1	Model 2	Model 3
*A priori* combinations*			
Low-risk level: Three items among age ≥85years, male gender, polypharmacy† and non-use of home services‡	**0.82** [0.74; 0.92]	**0.80** [0.71; 0.91]	1.00(Ref)
	(**0.001)**	(**0.001)**	
Intermediate-risk level: History of falls§, or temporal disorientation, or (age ≥85years + male gender + polypharmacy† + non-use of home services‡)	1.06 [0.95; 1.18]	1.06 [0.95; 1.19]	**1.20** [1.05; 1.36]
	(0.325)	(0.323)	(**0.006**)
High-risk level: history of falls§ + temporal disorientation	**1.27** [1.09; 1.49]	**1.29** [1.09; 1.51]	**1.44** [1.20; 1.73]
	(**0.003)**	(**0.002**)	(**<0.001**)
Newly identified combinations			
Low-risk level: polypharmacy† + (non-use of home-help services‡ or age ≥85years or history of falls§) + (absence of temporal disorientation)	0.93 [0.82; 1.06]	0.90 [0.79; 1.02]	1.00(Ref)
	(0.291)	(0.104)	
Intermediate-risk level: combinations other than low- or high-level combinations	0.91 [0.81; 1.02]	0.93 [0.82; 1.04]	1.05 [0.91; 1.19]
	(0.096)	(0.208)	(0.519)
High-risk level: temporal disorientation + polypharmacy† + (history of falls§ or (age ≥85years or non use of home help services‡))	**1.40** [1.17; 1.68]	**1.43** [1.19; 1.72]	**1.47** [1.20; 1.81]
	(**<0.001**)	(**<0.001**)	(**<0.001**)

Model 1: Unadjusted model.

Model 2: Model adjusted on organ failure and place of life.

Model 3: Low risk of prolonged length of hospital stay used as reference, with adjustment on organ failure and place of life.

*: Combinations described previously (Beauchet et al. J Emerg Med. 2013; 45: 739–45).

† : Defined by a number of drugs taken per day above 4.

‡ : Living alone without using any formal or informal home services and social help.

§ : In past 6 months.

¶ : Inability to give the month and/or year.

Hazard ratio and p-value significant (i.e., <0.05) indicated in bold.

**Table 4:** Cox regression models showing the association between the length of hospital stay (dependent variable) and combinations of brief geriatric assessment items (independent variables) separated into three risk-levels (i.e., low risk, intermediate risk, and high risk of prolonged length of hospital stay) with separated models for reasons for admission to emergency department (n = 1254).

Combinations of the 6-item brief geriatric Assessment stratified in three levels of risk (low, intermediate, high)	HR [95% CI] (P-Value)*				
	Cardio vascular disease	Respiratory disease	Digestive disease	Neuropsychological disease	Other diseases
*A priori* combinations†					
Low-risk level: three items among age ≥85years, male gender, polypharmacy‡ and non-use of home-help services§	1.00(Ref)	1.00(Ref)	1.00(Ref)	1.00(Ref)	1.00(Ref)
Intermediate-risk level: history of falls, or temporal disorientation#, or (age ≥85years + male gender + polypharmacy‡ + non-use of home-help services§)	**1.17** [1.04; 1.32]	**1.19** [1.06; 1.35]	**1.18** [1.04; 1.33]	**1.17** [1.04; 1.32]	**1.21** [1.07; 1.37]
	**(0.012)**	**(0.004)**	(**0.008**)	(**0.010**)	(**0.003**)
High-risk level: history of falls + temporal disorientation#	**1.39**[1.17; 1.65]	**1.44** [1.21; 1.70]	**1.41** [1.19; 1.67]	**1.40** [1.18; 1.65]	**1.46** [1.22; 1.74]
	(**<0.001**)	(**<0.001**)	(**<0.001**)	(**<0.001**)	(**<0.001**)
Newly identified combinations					
Low-risk level: polypharmacy‡ + (non use of home help services§ or age ≥85years or history of falls) + (absence of temporal disorientation)	1.00(Ref)	1.00(Ref)	1.00(Ref)	1.00(Ref)	1.00(Ref)
Intermediate-risk level: combinations other than low- or high-level combinations	1.05 [0.92; 1.19]	1.03 [0.91; 1.18]	1.04 [0.92; 1.19]	1.05 [0.92; 1.19]	1.04 [0.91; 1.18]
	(0.509)	(0.614)	(0.520)	(0.484)	(0.609)
High-risk level: temporal disorientation# + polypharmacy‡ + (history of falls or (age ≥85years + non-use of home-help services§))	**1.47** [1.19; 1.80]	**1.48** [1.20; 1.81]	**1.48** [1.20; 1.82]	**1.47** [1.20; 1.81]	**1.48** [1.21; 1.82]
	(**<0.001**)	(**<0.001**)	(**<0.001**)	(**<0.001**)	(**<0.001**)

All models used low-risk level as reference value.

*: adjustment on place of life.

† : Combinations described previously (Beauchet et al. J Emerg Med. 2013; 45: 739–45).

‡ : Defined by a number of drugs taken per day above 4.

§ : Living alone without using any formal or informal home services and social help.

¶ : In past 6 months.

# : Inability to give the month and/or year.

Hazard ratio and p-value significant (i.e., <0.05) indicated in bold.

**Figure 2:**
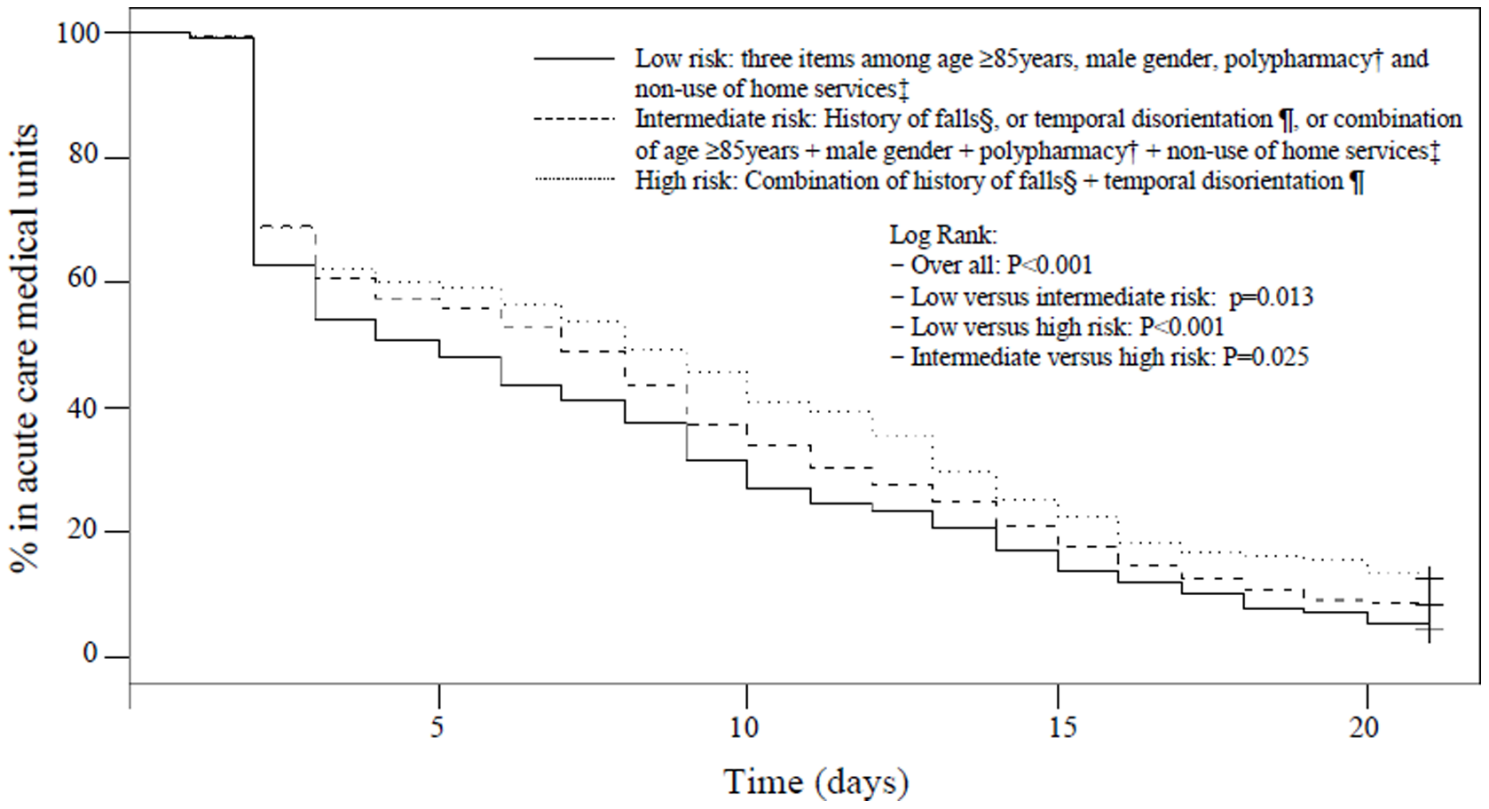
Kaplan-Meier estimates of the probability of discharge from acute care medical units among older inpatients (n = 12,054) using a priori* combinations of the 6 items of the brief geriatric assessment. *: Combinations described previously (Beauchet et al. J Emerg Med. 2013; 45: 739–45). †: Defined by a number of drugs taken per day above 4. ‡: Living alone without using any formal or informal home services and social help. §: In past 6 months. ¶: Inability to give the month and/or year.

**Figure 3:**
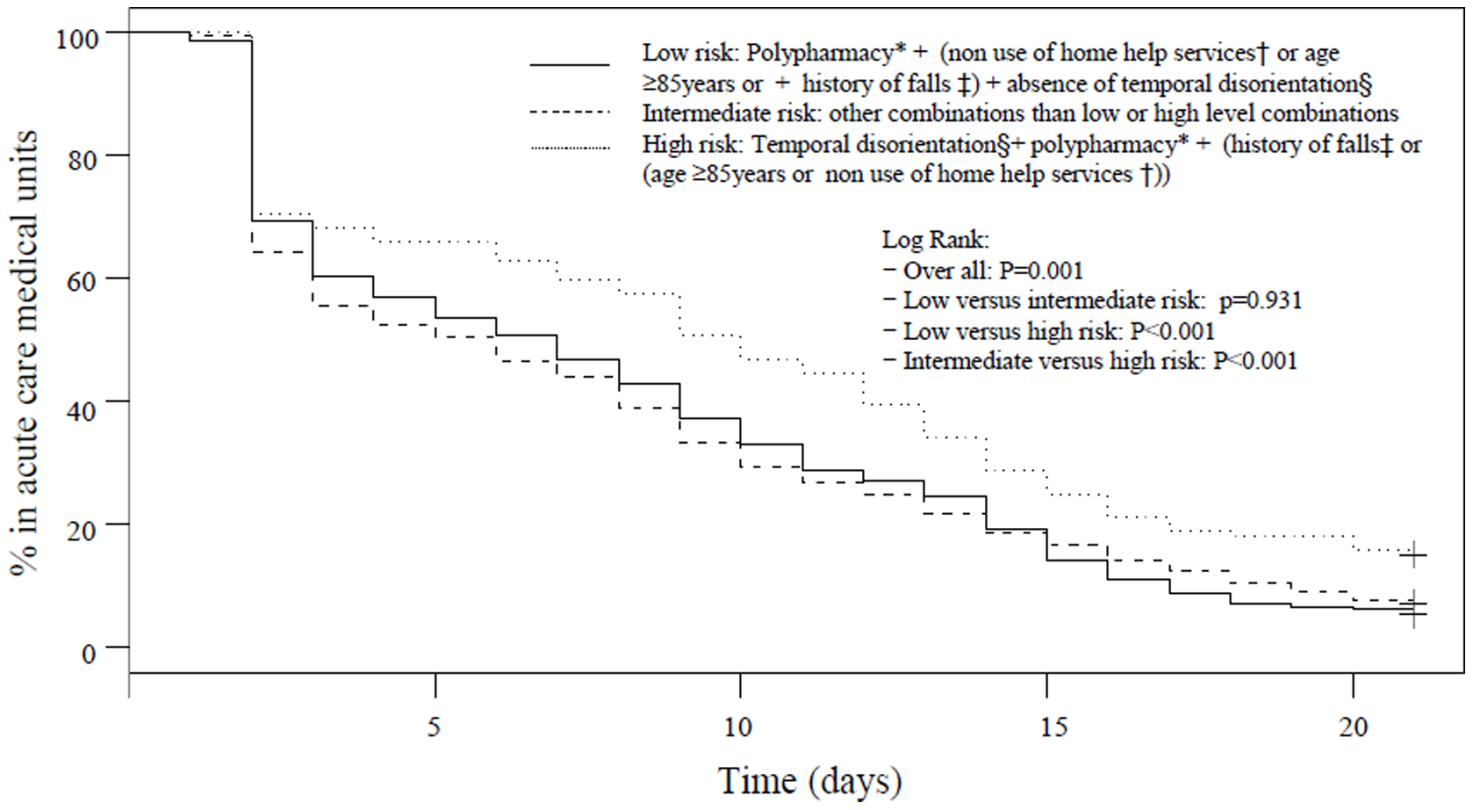
Kaplan-Meier estimates of the probability of discharge from acute care medical units among older inpatients (n = 12,054) using newly identified combinations of the 6 items of the brief geriatric assessment. *: Defined by a number of drugs taken per day above 4. †: Living alone without using any formal or informal home services and social help. ‡: In past 6 months. §: Inability to give the month and/or year.

Generated Kaplan-Meier plot of the probability of discharge from acute care medical units is reported Figures 2 et 3. While using *a priori* combinations of BGA items (Figure 2), comparison of survival curves showed that inpatients considered at low risk of prolonged LHS were faster discharged from acute care medical units than those in intermediate (P = 0.013) and high-risk groups (P<0.001). Furthermore, patients in intermediate risk of prolonged LHS were also faster discharged from acute care medical units than those in high-risk group (P = 0.026). The new classification showed similar results (Figure 3), except for the comparison of discharge between patients in low-risk group and those in intermediate-risk group which was not significant (P = 0.931). Comparison of survival curves showed that inpatients considered at low risk of prolonged LHS were faster discharged from acute care medical units than those in high-risk group (P<0.001). Furthermore, patients with intermediate risk of prolonged LHS were also faster discharged from acute care medical units than those in high-risk group (P<0.001).

## Discussion

Our results show that combinations of BGA items were significant risk factors for prolonged LHS in geriatric patients admitted to ED and discharged to acute care medical units. However, the prognostic value for prolonged LHS of these combinations of BGA items was poor, whatever the BGA items or their combinations used.

The main findings of our study is the discrepancy between the fact that combinations of BGA items were risk factors for prolonged LHS and their poor prognostic value for this endpoint. Limitations of risk factors as a prognostic tool have been previously reported [21]–[23]. The conclusions of these publications was that risk factors should exhibit a much stronger association with the studied endpoint (e.g., prolonged LHS in our case) than they ordinarily show in epidemiological studies if it is to provide a basis for prediction in individual patients. Our results are, therefore, in total concordance with this assumption. Indeed, even if HR of certain combinations of BGA items were significant risk factors for prolonged LHS, the magnitude of their values was low below 2, which is consistent with a poor prognostic value for prolonged LHS. This apparent paradoxical result (i.e., a significant risk factor having a poor prognostic value) is in relation with the fact that a risk factor is assessed by comparing the risk of studied outcome at each end of the distribution of the risk factor. While using this statistical approach the effect of being highly exposed to the factor is compared with the effect of being slightly exposed. Thus, comparison is done on jointly exclusive groups of individuals. The main consequence of this approach is that the group of individuals in the middle of distribution, which represents the majority of individuals, is ignored. Because the aim in screening tool is to identify a group of individuals with a high risk relative to everyone, there is necessary a discrepancy between to be a significant risk factor associated with the studied outcome and to be a poor prognostic risk factor for this outcome.

Combinations of BGA items were significant risk factors for prolonged LHS among studied geriatric inpatients. The strength of this association was greater when specific items were combined underscoring a stronger association while using newly identified combinations compared to *a priori* combinations [16]. This result may be explained by the fact that the BGA items, and specifically the combinations defining the high-risk level, are markers of frailty, which is a status previously identified as a risk factor for prolonged LHS [16], [17], [5]. Frailty in older adults usually refers to a physiological vulnerability medical that expose to adverse health, functional and social outcomes [24]. Despite the lack of consensus on the definition of frailty, there is a broad agreement that frailty is characterized by an accumulation of deficits related to a combined action of aging and morbidities [25]–[27]. Under the action of the morbidities and/or ageing that generate disabilities, this dynamic process, occurring from reduced physiological resources, results in impaired adaptation to stress such as a hospitalization. As a consequence frail older adults are more prone to prolonged LHS than non-frail older ones [16], [25]–[29].

Our results also show that the risk of prolonged LHS did not increase in a simple cumulative way according to the combination of items. Considered separately, older age, male gender, polypharmacy and the absence of home-help services have been previously associated with prolonged LHS [10], [16], [28]–[30]. The highest risk was not reported with the combination of all 6 items. Moreover, high risk of prolonged LHS was found with several combinations rather than one. This results is probably the consequence of a complex interplay of BGA items which are direct or indirect markers of geriatric syndromes, morbidities or functional decline, and where the interaction between medical and change of the environment of an individuals (i.e., hospital and more precisely acute care unit in our study) exposes patients to a major risk of prolonged LHS [16], [28], [29]. Indeed, the latter parameter underscores the inability of changes of frail individuals due to poor physiological resources. In addition, the heterogeneity of health conditions among geriatric population may also account for the various combinations related to a high risk of prolonged LHS [2], [8], [16].

Among the BGA items, we confirmed in the studied sample of inpatients that the history of falls and temporal disorientation were strongly related to prolonged LHS, and thus were part of most combinations defining the high risk of prolonged LHS [16]–[18]. In addition, the risk of prolonged LHS increased when both these items were associated. Falls usually cause and/or result from gait and/or balance disorders, which are themselves identified risk factors for prolonged LHS [31]–[33]. This specific association has been underscored using Timed Up & Go (TUG), which is a basic assessment of functional mobility and measures the time needed to rise from a chair, walk 3 meters, turn around and return to a seated position [33]. It has been reported that longer TUG was a marker of prolonged LHS in older patients [10], [18]. Furthermore, the association between temporal disorientation and prolonged LHS may be explained by the fact that temporal disorientation is a surrogate measure of severe cognitive disorders, either acute (i.e., delirium) and/or chronic (i.e., dementia) [17). Because these both cognitive disorders have been identified as risk factors for prolonged LHS, they could explain the prolonged LHS reported in our study [10], [18]. Our results also highlighted that the risk of prolonged LHS increased when history of falls was combined with temporal disorientation. This is in concordance with previous results showing that individuals combining cognitive decline and mobility disorders were more at risk of adverse outcomes than those having only one disorder, and that LHS increases with the accumulation of risk factors [18]–[20]. Finally, this strong association of history of falls and temporal disorientation with prolonged LHS may be explained by the fact that both these disorders are common ways of expression of diseases, whatever their nature. Older adults' health and functional status is heterogeneous because of the various cumulative effects of chronic diseases and physiologic decline, contributing to a vicious cycle of increased frailty, the frailty being defined as a clinically recognizable state of increased vulnerability [24]–[27]. The expression of this vulnerability is often a decrease in gait and cognitive performance, the clinical symptoms of this decline being falls and temporal disorientation [24], [25].

The poor prognostic value of the 6-item BGA is in concordance with results of previous studies exploring the predictive value of clinical parameters for the occurrence of adverse health events in older adults. Prognostic value of prior tools, such as the Identification of Seniors At Risk (ISAR) score and the triage risk stratification tool (TRST), which are the main tools validated in ED users aged 65 years and older, have been examined [34], [35]. Like our results, it has been reported that these tools have a poor prognostic value for adverse health outcomes in ED (i.e., death, institutionalisation, readmission) [34], [35]. Developing one single tool to predict LHS remains a complex challenge but an objective to reach. A perspective for the prediction of frailty-related adverse outcomes such as prolonged LHS could come from non-linear statistical methods [36]–[38]. For instance, artificial neural networks (ANNs) are data analysis tools that have been developed to overcome limitations of linear models [36]. Because they apply non-linear statistic to pattern recognition, ANNs are particularly adapted to “chaotic” behavior like frailty-related adverse outcomes. Nowadays, the advance of ANNs combined with improvement of computers technology open new perspectives in diagnostic support aids for helping physicians to take the best decision for their patients [37], [38].

Some limitations of this study need to be considered. First, participants were included from a single centre and, thus, they were probably not representative of all older adults admitted to ED. Secondly, although we were able to control for many characteristics likely to modify the LHS, residual potential confounders might still be present. We limited this confounding bias by adjusting our results on organ failure and place of life because these covariables are strong determinants of prolonged LHS [17]–[18]. Third, there was a potential recall bias about the history of falls, which is well known in the elderly. Falls are usually underreported because of a cognitive decline of fallers who forget to report the falls, and depends on the occurrence of fall-related adverse health outcomes [39].

In conclusion, combinations of 6 BGA items were significant risk factors for prolonged LHS with a risk that did not increase linearly with the accumulation of items, underscoring that some specific combinations of BGA items were greater risk factors than others. Unfortunately, their prognostic value was poor. Further research is required to improve the prognostic value of clinical tools for the identification of adverse events among older adults hospitalized in medical and surgical care units. A perspective may rely on the use of ANNs.
